# Teaching anti-racism at the bedside: perspectives from patients and clinician educators

**DOI:** 10.1186/s12909-025-08312-2

**Published:** 2025-12-02

**Authors:** Samantha XY Wang, Matthew Burke, Shay Taylor, Taylor Hollis, Nicole K. Corso, Cati Brown Johnson, Sonoo Thadaney Israni, Donna M. Zulman

**Affiliations:** 1 Division of Hospital Medicine, Department of Medicine, Stanford School of Medicine, Stanford, CA USA; 2 Meharry Medical College, Nashville, TN USA; 3 Howard University College of Medicine, Washington, DC USA; 4 University of Alabama at Birmingham, Heersink School of Medicine, Birmingham, AL USA; 5 Division of Primary Care and Population Health, Department of Medicine, Stanford School of Medicine, Stanford, CA USA; 6 Department of Medicine, Stanford School of Medicine, Stanford, CA USA; 7 300 Pasteur MC, Stanford, CA 5158, 94503 USA

## Abstract

**Background:**

Anti-racism medical education is essential for addressing health disparities and improving patient care. This study examined patient perspectives onengaging in anti-racism discussions at the bedside and explored barriers and facilitators facedby clinician educators when teaching anti-racism concepts to clinical learners. examined patient perspectives on engaging in these discussions at the bedside.

**Design:**

This qualitative study utilized focus group discussions withpatients from underrepresented communities and semi-structured interviews with clinicianeducators.

**Participants:**

Patients (*n* = 17) were recruited from a Virtual National Community Advisory Board, comprising patients and clinicians caring for Black patients in Leeds, AL; Memphis, TN; Oakland, CA; and Rochester, NY. Clinician educators (*n* = 10) were recruited from two academic medical centers in California and Alabama.

**Approach:**

Patient focus groups introduced participants to teaching scenarios around bias and structural racism in clinical decision making, and invited response and discussion focused on perceived comfort as a patient, provider-patient rapport, and sense of inclusion in the teaching moment. 1:1 clinician educator interviews focused on knowledge of structural racism, experiences discussing anti-racism, and perceived barriers and facilitators when teaching these concepts in clinical settings.

**Key results:**

Patients expressed interest to engage in bedside discussions about their racialized experiences, emphasizing the importance of patient narratives in these conversations. Both clinician educators and patients agreed on the importance of including patient stories and voices in bedside teaching on anti-racism. Clinician educators identified significant barriers to teaching anti-racism at the bedside: systemic challenges (e.g. time constraints), lack of structural support, personal discomfort, and fear of retaliation. Facilitators included supportive learning communities and structured curricula.

**Conclusions:**

This study highlights the potential to enhance anti-racism education in medical training by incorporating patient voices into bedside teaching. This approach empowers patients and enriches clinician educators’ understanding of relevant racialized experiences. Future research should focus on the practical implementation of these discussions in clinical environments and their impact on patient outcomes.

**Supplementary Information:**

The online version contains supplementary material available at 10.1186/s12909-025-08312-2.

## Background

Academic medical centers have increasingly developed and implemented anti-racism curricula for students, trainees, and faculty over the past 5 years [1–7]. Racism directly drives inequities, contributing to delays in diagnosis and treatment, higher rates of medical errors, and worse health outcomes for marginalized patients [8–10]. Anti-racism, defined as the active process of identifying, challenging, and changing policies, practices, and structures that perpetuate inequities in health care, provides a necessary framework to confront these harms [11]. Despite educational innovations, significant barriers remain to integrating anti-racism discussions seamlessly in the clinical environment. Faculty often lack formal training, institutional support, and confidence in managing sensitive conversations, fearing that introducing this topic may provoke discomfort for learners and patients [12]. Resultingly, discussions about these complex issues of systemic racism, implicit bias, and health disparities are too frequently pushed aside, missing a vital opportunity to link classroom theory with real-world practice [13]. 

These challenges echo mid-20th century concerns about bedside teaching itself, when educators feared that teaching at the patient’s bedside might compromise patient dignity, contribute to psychological trauma, or negatively affect patient outcomes [14]. Yet decades of research have shown that well-structured bedside teaching actually enhances diagnostic skills, builds rapport, promotes shared decision-making, and deepens patient-centered care [15–18]. Patients often welcome participating in the teaching experience [19]. Success requires educators be trained in patient consent, patient dignity, and active patient participation in the teaching process [19].

Most existing anti-racism teaching remain confined to classrooms, workshops, or reflection series, isolating learners from the very patients they aim to serve. Conducting these conversations away from the bedside risks depersonalizing minoritized patients and silencing their lived experiences. By bridging patient narratives with theoretical foundations, bedside educators have an opportunity to foster meaningful dialogue, deepen learners’ understanding of structural racism, and cultivate empathy by center patient voices in clinical teaching. In this study, we convened a community advisory board of under represented patients to explore racialized experiences navigating the healthcare system, prior exposure to bedside anti-racism conversations, and gather their reflections on teaching scenarios around bias and structural racism in clinical decision making and its effects on patient comfort, provider-patient rapport, and sense of inclusion in the teaching moment. We also invited clinician educators to share their perspectives on barriers and facilitators to teaching anti-racism at the bedside.

## Methods

We conducted a qualitative study with a community advisory board of patients from historically underserved communities and with clinician educators at two academic medical centers. The study was approved by the Stanford institutional review board (Protocol #60646). Informed consent was obtained from all participants prior to data collection. This study was conducted in accordance with the ethical principles outlined in the Declaration of Helsinki.

### Community advisory board: patient focus groups

Patients were recruited from the Presence 5 Virtual National Community Advisory Board, via an email invitation distributed to the board’s listserv. Participation was voluntary and interested members contacted the study team to enroll. The Community Advisory Board includes patients and clinicians from clinics that predominantly serve Black patients in Leeds, Alabama; Memphis, Tennessee; Oakland, California; and Rochester, New York [20]. Each focus group was held over video conferencing, lasted 90 min and audio-recorded. Patient focus groups had 3–8 participants and followed a structured format (Supplemental 1). At the start of each focus group, participants received background information on how racial bias influences health care testing, treatment, and clinical decisions, along with the aims of the study and an established framework for teaching anti-racism with clinical learners. Facilitators defined the clinical learning context as environments where trainees learn through direct patient interactions (e.g., wards, clinics, bedside) and reviewed the roles of clinical team members. Participants provided initial feedback on the framework before reviewing two cases written at a sixth-to-eighth grade reading level: (1) a scripted case on kidney health and race correction in creatinine calculation and (2) an animated case highlighting racial bias in diagnosing skin conditions in patients with darker skin tones. After each case, participants shared impressions and reflected on hearing such content from physicians at the bedside, including ways to integrate their own narratives. Discussions focused on patient comfort, rapport and trust, and inclusion in the teaching process. Focus groups were conducted by Black facilitators racially concordant with most participants (all but two identified as Black). Participants received a $100 gift card for completing the focus group.

## Clinician educator interviews

Clinician educators were recruited using a convenience sampling approach, drawing on existing institutional partnerships and professional networks at two academic medical centers. The interview guide focused on: (1) clinician knowledge of how racism and racial bias impacts patient care and health outcomes; (2) clinician experience of discussing bias, race and racism in clinical decision making with clinical learners; (3) barriers and facilitators for these conversations in the clinical learning environment. Interviews were conducted by study members (M.B. and S.W.) between July 2021 and August 2023 using video-conferencing software. The 1:1 interviews (30–45 min), were audio-recorded, downloaded, and auto-transcribed. The auto-generated transcripts were manually reviewed for accuracy by three study members (S.W., S.T., and M.B.) who listened to the original audio recordings and compared them line-by-line with the transcripts to identify and correct errors. The interview guide is included in Supplemental 2.

### Data analysis

We used a combination of inductive and deductive thematic approaches to analyze clinician interviews and patient focus group comments. Each interview and focus group transcript was independently analyzed by at least two members of the research team to ensure consistency and depth in coding. Throughout the analysis process, the team met bi-weekly to review and discuss thematic codes. Coding discrepancies were resolved through group dialogue and consensus, resulting in the iterative refinement of a comprehensive final codebook [21]. This approach resulted in identification of emergent themes while ensuring alignment with our predefined research objectives. We used Dedoose software to manage and code the data.

## Results

Seventeen individuals participated in the patient focus groups, comprising 16 patients (15 Black, 1 LatinX), and 1 white clinician. The clinician educator interviews included 10 internal medicine physicians across two academic practice settings: Stanford School of Medicine (*n* = 5), and University of Alabama at Birmingham School of Medicine (*n* = 5). Clinician educators practiced in a range of settings in suburban and urban locations. Among clinician educators, 5 were men and 5 were women; Six identified as White, 2 as Asian, and 2 as Black; 3 graduated from residency within the past 5 years, 3 within the past 5 to 10 years, and 4 more than 10 years ago..

### Qualitative findings

#### Community advisory board focus groups: patient perspectives about bedside teaching with an anti-racism focus

Focus group discussion identified that many patients agree that the most crucial aspect of navigating conversations about anti-racism in healthcare is the rapport between the patient and clinician. Many patients expressed a preference for race-concordant clinician-patient relationships. As one patient articulated, “Having an African American physician changes things totally; how I communicate my comfort level and everything else… but having to choose your words with a [non-Black] physician, you know you do that extra step.” This sentiment was echoed by other patient participants who noted that a shared cultural identity fosters a deeper understanding and facilitates better communication: “If my doctor is African American…it’s like they’re looking out for their people, and I think that’s with any culture.”

Further supporting the importance of race-concordance, patient participants also discussed negative experiences of race discordance, revealing significant barriers to effective rapport-building. One patient recounted an encounter with a race-discordant clinician, stating, “I felt like she was giving me suggestions that were unrealistic for me.” Furthermore, the fear of harm in healthcare settings was a recurring theme: “We were raised to fear the doctor; there’s fear that white doctors are here to hurt us, so we do home remedies that don’t always work.”

When discussing the impacts of racism and bias on their healthcare experiences, patient participants recounted feelings of disempowerment, lack of knowledge, and disbelief from the healthcare system. One shared a distressing experience: “I was at an eye appointment and the doctor saw something unusual. I didn’t realize it was a teaching institution, and he kept inviting people to look at my eye…. I was terrified because I thought I had a tumor, and he finally saw the terrified look. It wasn’t until he saw my discomfort that he remembered his bedside manner.” Another recalled negative interactions with previous clinicians, expressing concerns such as, “Are they looking at me when they speak to me?” and “I went to a new doctor for a check-up, and he did not even do an exam. He did not even touch my Black skin.”

Despite these challenges, some patients described facilitators that can enhance rapport in race-discordant relationships. Successful connection-building often relied on the clinician’s holistic understanding of the patient as a person. One participant emphasized, “It’s just really a matter of do you hear me, do you see me, do you really understand what I’m going through in addition to my condition I’m telling you about?” Additionally, there was a desire for more transparency and patient empowerment through knowledge: “Address all my concerns. I want to know. I want to explore every option and opportunity. For treatments, I want to participate intelligently in my treatment plans.”

### Community advisory board focus groups: patient advised strategies for teaching anti-racism at the bedside

Patients participating in focus groups were asked about the acceptability of hearing physicians discuss anti-racism in clinical decision-making at the bedside and how physicians could prioritize the patient narrative. Initially, patients were hesitant about the idea of discussing racism during bedside teaching. However, over the course of the discussion, patients became more curious and empowered. One noted, “For me, it would shock me if someone were to come in the room and start this conversation, because it has never happened before. But I would much prefer you coming in, however difficult the conversation may be, than to ignore it.” Another expressed a willingness to engage in the topic: “I feel like I would not mind students entering my appointment or conversation, especially this learning method. I know that it’s kind of building a better tomorrow.” One poignantly stated, “Let’s roll up our sleeves and have the conversation. Let’s do that in a very respectful way, and I think that will definitely resonate with a lot of people because, again, the conversations are not being had out in the open. We’re having them in the breakrooms, on the phone sidebars, but we’re not really having those hard conversations where they need to be had in the room… But how enlightening would that be? To show them like, there are doctors that don’t look like you, that really do care about you.” Patients also provided suggestions and strategies for educators to promote patient engagement, comfort, and acceptability when discussing race at the bedside (Table 1).

**Table 1:** Strategies for teaching Anti-Racism at the Bedside, informed by focus group discussions with patients

Steps	Strategies and Goals	Quotes
Prepare	Describe context privately with the patient	Patient: “I would want to hear from the doctor: we are going to bring this team in, one of the things are want to make sure we are doing better in how to serve our patients better from a cultural sensitivity and competency way.” Patient: “Let me know what is going to happen *before* you bring your team in.” Patient: “I also agree that it would potentially be awkward but with some warning, if the patient were told ahead of time, and if they were told that they were asked for their ideas: *we are looking for your advice and ideas for how race has affected you*, it would allow me to think about how I would phrase things.”
	Establish trust	Patient: “What kind of relationship rapport do I have with that clinician? What’s our comfort with each other? and if the answer is, we aren’t there yet, then we need to take a different approach”
	Prioritize patient comfort and well-being	Patient: “If you were in pain and the doctor came in, that’s an inappropriate time to even begin to ask for me to contribute anything to this discussion.”
	Display visible allyship	Clinician: “Such as wearing a Black Lives Matter badge or pin on your white coat that shows you’re an ally.”
Prepare	Ask for permission in private	Patient: “I would ask the patient for permission to understand…what is in it for you as a patient? So, the medical team can better understand your cultural, mental, emotional needs. As opposed to all of them coming from the door and doing their diagnostic overview, and I am sitting there like a bird in a cage, not understanding anything.”
Empower Patient Narratives	Explore patient narratives and perspectives first	Patient: “But in terms of having that discussion about race, I think as long as there’s a mutual agreement, then it’s okay to talk about it. Then there’s a space for patients to provide their perspectives and their experiences. So, it’s not just a one-way street… This is a collaborative process here.” Patient: “Ask the patient: what is your perspective of what’s happened to you? …And what’s your perception? It sets the stage and gives the power to me… because a lot of times you feel so powerless in whether that’s in the hospital, inpatient, outpatient. I’m handing my power off to somebody else. But if you give me my power back with just a simple prompt of okay, you start us off with your perspective. I’m like, oh, I can honestly tell you any of the millions of encounters [of bias] that I’ve had with myself and my daughter.”Patient: “I would agree that having the patient involved in the conversation and the discourse is critical for their own self-healing.”
	Encourage patient to see themselves as teachers	Patient: “Flip it so the patient is the teacher and not the doctor.”Patient: “I personally would be comfortable learning right along with learners…because it would be like a tool for self-advocacy for myself with that knowledge… but only if you invite the patient into the conversation.”
	Don’t assume shared experiences	Patient: “It’s really about the language on how you are delivering it… you just have to be very mindful [because] it could come off as “they’re stereotyping me.””
Mindful Communication	Avoid medical jargon	Patient: “They might feel disempowered because of the medical jargon and all the medical talk. So have them speak first and early.” Patient Participant: “If the terminology is so high level, that they’re like, what is this this is Greek, it’s gonna go in one ear and out of the other, right?”
	Address the patient as respectfully	Patient: “Make sure that the pronouns that are used do not alienate the patient. Try to avoid using third person. Look at the patient and use direct second person pronouns, *you*, when you are talking with the person in front of you.”Patient: “Being more respectful of the patient, by using their name, Mrs. or Mr. Jones or Miss Thomas or whatever…that to me is critically important.”
	Transition thoughtfully into race discussions	Patient: “If I had sickle cell if [the doctors] were to say hey I really would like to speak with these students about how you know sickle cell something is more common in African Americans like that’s something that I would be understanding of.”
Facilitate Open Dialogue	Invite patient’s questions, reflections, and feedback from the patient	Patient: “I can imagine coming away from such a visit, then being able to really think on it on my own terms and then discovering, oh, I need to know a little bit more information about this or I have more questions, or I have more feedback…” Patient: “After talking about the facts of the history racism in medicine ask [me] how does this make you feel what we just talked about? Does this resonate with you? Give me a chance to provide feedback on the situation. Again, it’s always about making sure the patient’s voice is recognized and heard.”

### Clinician educator interviews: perspectives on teaching anti-racism at the bedside

#### Knowledge of structural racism

Clinician educators recognized issues such as redlining, mass incarceration, racial profiling, voter restrictions, and the non-expansion of Medicaid and their impacts on health outcomes [18]. One clinician educator noted, “Many of the neighborhoods in Birmingham and surrounding suburbs are racially segregated due to decisions made in the 1950 s and 60 s, leading to disparities in neighborhood resources. Many of our African American patients come from under-resourced areas, affecting both their access to healthcare and their healthcare decisions.” Another clinician educator highlighted the existence of “food deserts, characterized by a prevalence of calorically dense foods in Black communities, resulting in higher obesity rates.”

Clinician educators readily recalled instances of racism in healthcare, often citing disparities such as “pain control in patients with sickle cell crisis,” unequal access to COVID-19 therapies, and insufficient diagnostic workups or treatment for pain in patients from marginalized identities.

### Barriers to teaching anti-racism in the clinical environment

Clinician educators expressed feeling unprepared when teaching and discussing bias in healthcare because they lack the skills. Three themes emerged: challenges within the learning environment, a perceived lack of authority, and fear of potential consequences. Table 2 features select clinician educator quotes that underscore the multifaceted challenges when attempting to incorporate anti-racism into clinical education. Participants noted that the lack of time, competing demands, and absence of standardized tools to engage in anti-racism conversations with clinical learners presented as learning environment challenges, with one educator stating “There are so many topics that are important to teach students and residents. At the end of the day, they compete as to what learners and teacher prioritize as topics of interest.” Many barriers related to lack of authority, with sub-themes of feeling untrained, lacking the words to start these conversations, having no previous experience of racism, being unclear on what actional steps could be taken to promote change, and worrying about being unable to respond to learners or patients. Within Fear of Consequences, educators were worried of being misconstrued by learners, experiencing retaliation from learners or creating mistrust from patients, and avoided difficult conversations.

**Table 2:** Clinician educator-reported barriers to teaching anti-racism in the clinical environment

Themes	Sub-Themes	Exemplar Quotes
**Learning Environment Challenges**	Lack of timeCompeting demands	“There are so many topics that are important to teach students and residents. At the end of the day, they compete as to what learners and teacher prioritize as topics of interest.”
	Absence of standardized tools	“A lot of us who are now in more senior roles did not have this these sorts of discussions or frameworks.”
Lack of Authority	Feeling untrained	“Race feels like a big subject to broach out of the barn. I try to find other ‘ins’ to talk about this, such as religion or bias.”
	Lacking the words to start these conversations	“Having the technical know-how and verbiage to use because we want to be sensitive and intentional about the language we use.”
	No previous experience of racism	“When I teach about something, I like to have expertise in that area. As a white guy in America, I feel that I don’t have expertise in racism.”
	Unclear on actionable steps for change	“What do we do with this information? Do we have actionable ways to change our behaviors and ways? It seems easier to describe the events but harder to say what we will do about it.”“People have, they’ve sat through a lecture about unconscious biases, and they realize they have unconscious biases and then they don’t know how to apply that into their actual practice of seeing patients.”
	Worrying about inability to respond to learners or patients	“The first step is to have that knowledge to be able to answer the difficult questions and help others address racism.”“As faculty, you have a feeling that you should feel like you know everything.”
Fear of Consequences	Being misconstrued by learners	“Being a white male, most privileged, you may come across wrong.” “That feeling of me getting up on my soapbox is a bit of a white savior complex…and it makes me uncomfortable. Am I putting my nose into a problem that doesn’t really need me?”
	Fear of retaliation from learners or creating mistrust in patients	“The last thing I’d want to do is to be off putting to learners or even to patients.”“If you say something incorrectly or didn’t mean to imply, it could harm you or your position in the future.”
	Avoidance of difficult conversations	“So many of patients are exhausted from living in systems that are racist. I generally avoid reminding them of this.”“It’s awkward to discuss because if you have a diverse team, you don’t know how they may respond to a reminder of how hard it is to be black, or Latina, a refugee, or a migrant. And you don’t know how to do it sensitively because you may not identify as part of that group.”

### Facilitators to teaching anti-racism to trainees in the clinical environment

In analyzing facilitators to teaching anti-racism at the bedside, three themes emerged: being part of a learning community; having a teaching toolkit; and personal strategies in resilience and reflection (Table 3). Collectively, these facilitators can empower clinicians to engage learners meaningfully in anti-racism education. When discussing a learning community as a facilitator, sub-themes of feeling not alone in this work, being supported by the institution, and flattening learning hierarchies compelled educators to continue this work. One educator noted, by “having institutional champions in this work all over the country, and so it’s a network of people working together, who value this work and keep it moving forward.” Additionally, the sub-theme of receiving positive feedback from learners was a motivating factor for educators, “just knowing that people want it, is helpful…” Educators also reported having an anti-racism teaching toolkit was a facilitator, including strategies for prioritizing time for this topic, setting expectations, and access to teaching scripts and structured frameworks. Educators noted that intentionality is needed in making time for these conversations, “we purposefully set time after clinic to talk about it.” Finally, educators also reported personal strategies of resilience and reflection, including maintaining a growth mindset, embracing discomfort, embracing personal narratives, and repeated practice, “you just got to do it. But if we keep doing it, it’s going to get easier and we’re going to find our words and find our stories.”

**Table 3:** Clinician educator-reported facilitators to teaching anti-racism in the clinical environment

Theme	Sub-Themes	Quotes
Learning Community	Not being alone in doing this work	“You can’t be the only voice in a team that’s advocating for equity. There needs to be a collective force towards this.”
	Institution values anti-racism work	“…having institutional champions in this work all over the country, and so that it’s a network of people working together, who value this work and keep it moving forward.”
	Flattening the hierarchy in traditional learning team structures	“By owning my lack of experience with this topic, it has opened me up to the opportunity to learn from others on our team and learn from others’ experiences and rather than just sharing with the team myself.”“I have done it wrong for many years and learned from those wrong doings. What I have learned from my colleagues is being able to own my biases and normalize the presence of them, so we can have a frank discussion with learners, so they see that the attending does not always have the right answer.”
	Receiving positive feedback from learners	“We got a lot of positive feedback from residents, and I ended up starting a patient experience forum that brought patients in to discuss their experience with the healthcare system through the lens of race or disability or addiction.”“Just knowing that people want it is helpful…we did a survey, and this was something that residents asked for, not something we were forcing them to learn.”
Anti-Racism Teaching Toolkit	Prioritizing Time for Teaching Anti-Racism	“At our institution, we have added small moments at the end of the morning report for reflection on social determinants of health that affected the patient.” “We purposely set time after clinic to talk about it. I felt like we could have talked for hours on it, but we spent 20 min.”
	Setting Expectations	“Set the expectations and the agenda for the learning session so letting people know off at the very beginning… this is meant to educate not to point any fingers.”“The attending takes the lead in setting up the ground expectation and can encourage learners to recognize biases in order to improve care delivery. Being in that environment encourages people to speak up.”
	Accessing Teaching Scripts and Structured Frameworks	“Having an ability to take one topic that comes up frequently and then having a platform to start those discussions earlier. I use the example of diagnostic errors in patients with darker skin.”
Personal Strategies of Resilience and Reflection	Growth Mindset Embracing Discomfort	“The first time I started teaching at the bedside, I was super nervous. ‘Am I going to look like I’m not qualified?’ It took doing it a lot to get comfortable. The same is going to be true about teaching about racism and its effects on patients in our healthcare system. It’s going to be hard but if we keep doing it, it’s going to get easier and we’re going to find our words and stories.”
	Embracing personal narratives	“It is through our own personal experiences that we get to share our stories and impact the lives of those who are learning and training.”
	Repeated Practice	“You just got to do it… But if we keep doing it it’s going to get easier, and we’re going to find our words and find our stories.”

## Discussion

This study contributes to a growing body of literature advocating for robust faculty development programs in anti-racism training [22, 23]. To our knowledge, it is the first study to include patient perspectives on anti-racism teaching at the bedside, adding an important dimension to this conversation. We found that patients in this study expressed an interest to participate in race-related teachable moments and share their experiences to highlight the importance of patient narratives and drive systemic change. The gap between clinician teaching practices and patient’s expressed interest in participating highlights an opportunity to strengthen faculty development, enhance curricula, and promote more patient-centered engagement.

Historically, clinical and educational spaces have often marginalized patient voices, particularly around discussions of race and inequity, leading many clinicians to assume that such conversations might burden or distress patients. Our findings challenge that assumption. While some patients acknowledged that discussing such topics might be uncomfortable, there was a shared recognition that this discomfort is often necessary for driving meaningful change. Many patients expressed a preference for these conversations to take place in their presence, allowing them to contribute their unique perspectives. However, establishing trust and rapport with patients is essential before navigating these discussions, as it creates a safe space for open dialogue and mutual understanding [24, 25]. By inviting patients to participate in bedside teachable moments, clinician educators can enhance learner understanding and empower patients as partners in their own care and in broader systemic change efforts. Similarly, our findings suggest that anti-racism teaching can be successfully integrated into clinical care by employing a structured, thoughtful approach that centers the patient’s voice and experience. This shift in teaching practice requires disrupting traditional medical hierarchies, where attending physicians are often viewed as the ultimate authorities on knowledge. Inviting patients to contribute their narratives challenges the “clinical gaze” that frames patients as objects of diagnosis, and instead centers them as co-educators in learning [26]. This reorientation reflects principles of patient-centered care and narrative humility, making abstract concepts like structural racism more tangible for learners and create a more inclusive learning environment [27]. 

Our results also highlight variability in clinicians’ comfort and experience discussing anti-racism at the bedside, despite broad recognition of structural racism as a contributor to health inequities. This gap is attributable to individual (e.g. faculty and learner interests; faculty expertise), environmental (e.g. time, competing patient-care needs) and systemic barriers (e.g. curriculum resources, financial resources, institutional support) [28].

Despite the valuable insights gained from this study, several limitations should be acknowledged. First, the clinician educators interviewed were drawn from only two academic institutions, limiting the potential generalizability of our findings. The perspectives of educators from a wider range of institutions including community-based hospitals, rural, and non-academic settings may differ, especially in terms of institutional support, resources, and local patient populations. Second, the focus groups with patients were conducted in controlled settings that lacked the inherent pressures and time constraints of a clinical environment. This may not fully capture the feasibility and acceptability of initiating anti-racism discussions in real-time clinical spaces, where stressors such as patient acuity, workflow demands, and potential emotional distress for both patients and clinicians could influence the dynamics of these conversations. Future research could benefit from exploring these dialogues, examining how these factors impact the practical implementation of bedside anti-racism education and patient outcomes. Thirdly, our community advisory board participants may have a higher inclination than the broader patient population to discuss racialized experiences in healthcare, so their level of interest and engagement may not be generalizable.

This study underscores the importance of faculty development in facilitating anti-racism education, which raises questions about how medical institutions can best equip educators with the necessary skills. Training efforts should focus on teaching educators how to invite patient engagement during critical discussions about race and ensuring that patient perspectives are integrated into training modalities. Additionally, investigating how participation in learning communities focused on anti-racism and health equity influences clinician behavior and teaching practices over time would provide valuable insights into how to sustain engagement. Looking ahead, it is imperative that medical education evolves to not only address systemic inequities but also to empower future healthcare clinicians to be advocates for racial justice, ultimately improving patient care and outcomes for marginalized communities. This will ensure that these efforts are not one-off initiatives, but fundamental principles guiding medical education and practice.

## Supplementary Information

Supplementary Material 1

Supplementary Material 2

## Data Availability

The datasets used and analyzed are available from the corresponding author on reasonable request.
