# Supplementary material for: Teaching anti-racism at the bedside: perspectives from patients and clinician educators
Source: BMC Med Educ. 2025 Dec 2;26:43. doi: 10.1186/s12909-025-08312-2 (PMC12797735; doi:10.1186/s12909-025-08312-2)
Supplement: Supplementary file 1 — Supplementary Material 1 [file 12909_2025_8312_MOESM1_ESM.pdf]

5-Minute Moment for Racial Justice Clinician Educator Interview Guide Interview Guide Questions/Statements:

"Hello Dr. ___. We are very grateful for your participation in this interview for the "5-minute moment for racial justice". If you are unfamiliar with this research we are working to understand challenges in teaching about racial equity during bedside rounds. This interview portion should will be audio-recorded and everything you share will be kept confidential. I will have my videooff, and you are invited to turn off your video if you'd like as well but leave your microphone on. I will ask you some questions, please do your best to answer as thoroughly as possible. Before we get started, do you have any questions for me?"

1. **First question: what is your current position? (Can you tell me what your current clinical role is?)** a. Affiliation & Medical Center b. Patient population you care for

2. **What race or ethnicity do you most identify with? What gender do you most identify with?**

   The next few questions will be around personal experiences of race or racism, and also how you teach about this topic. If you don't want to answer, please just say, "I don't know" or "Not applicable" or "Next question."

3. **Have you ever previously experienced racism personally?** It's okay to take a few minutes to reflect. [Pause for answer] Can you tell me what happened?
   a. How did it make you feel?
   b. What did you do about it? Did you tell anybody else about it? Did it ever happen again?

4. **How do you think race or racial bias affects healthcare?** Can you give some examples or ways in which you've noticed this?

5. **Have you heard about social determinants of health?** (how or when did you come to learn about this)? Ie. is this something you learned more about in recent years? What about structural determinants of health? If so, how would you define/describe it?
   a. If the interviewee asks you what are social determinants of health refer to examples (5b). If the interviewee asks you what are structural determinants of health refer to examples (5c).
   b. "Some examples of social determinants of health include → Safe housing, transportation, and neighborhoods, Racism, discrimination, and violence Education, job opportunities, and income Access to nutritious foods and physical activity opportunities Polluted air and water Language and literacy skills."
   c. "Some examples of structural determinants of health include → governmental, law, and societal policies and norms, that may not be accounted for with SDOH. Zoning, access to insurance, and the food options in specific regions."

6. **Describe a time in the past 12 months where race or racial bias may have affected the medical evaluation or treatment of a patient you have cared for. What happened? Did you address this or bring it up with your patient?**
    a. If yes, ask them how did it go
    b. If no, ask why not

7. **Think back to working with medical students and doctors-in-training over the past 12 months. What experience have you previously had when it comes to talking to learners about race and racism in medicine?** What sparked these conversations?

8. **Do you recall bringing up race or racial bias specifically as a term or risk factor for this patient to your learners (residents and medical students)**
    a. If response includes an example with learners: did you talk about (debrief) this with your learners afterwards? Why or why not? How did that go for you and for your learners (learner response)?

9. **Can you describe some barriers to talking about race and racism with your learners?** (Please describe 3) → Would you like to elaborate on any of those and what happened?

10. **What would be helpful to remove some of these barriers or encourage you to speak more about race and racism and how it affects healthcare with your learners?**

11. **What inspired you to learn more or seek more research on this topic? (Possibly skip)**

Thank you for your participation! This concludes our interview. Your responses are very important and help us progress towards racial health equity. Are there any further questions you have for me?
