# Supplementary material for: Teaching anti-racism at the bedside: perspectives from patients and clinician educators
Source: BMC Med Educ. 2025 Dec 2;26:43. doi: 10.1186/s12909-025-08312-2 (PMC12797735; doi:10.1186/s12909-025-08312-2)
Supplement: Supplementary file 2 — Supplementary Material 2 [file 12909_2025_8312_MOESM2_ESM.pdf]

Stanford Welcomes CAB Members!

Facilitator: Hi my name is X, it's great to see everybody joining our focus group today. I'd like us to take the time to introduce ourselves. Please share your name and where you are from.

Defining racial justice and health care:
Facilitator: We're here to talk about race, racism, and racial justice in health care. We recognize that Medicine is not free from racial bias and many of the modern-day approaches, testing, treatment reflect bias. Your participation today will help us redefine with justice in health care looks like.

Our goal today is to create curriculum that allows us to talk to learners and patients to improve health care. The aims of this curriculum include: Teach the historical roots of racial bias in healthcare and its effects for Black patients; Reduce health disparities; Promote conversations about race and racial bias with doctors-in-training; Invite the patient to contribute to the teaching moment (with personal experiences, things they have heard from their peers, etc.)

The purpose of today's CAB meeting is: to give Feedback on teaching topics and how to talk about and teach about race and racial bias in healthcare in front of students and doctors-intraining; the Information will be used to develop anti-racism teaching practices and a new curriculum to promote health equity for Black patients; This meeting will be audio and video recorded.

So let's get started. What does this curriculum look like in action?

Defining Clinical Learning Environment

Facilitator: First we need to talk about the setting. It takes place in what we call "The clinical learning environment". This is a setting where a student or a learner can encounter a patient. It might be the emergency room, or a clinic office, or in the hospital. The learning often happens in teams. That's why you see a picture with a group of doctors – there's a head doctor called "the attending" who is the supervisor in charge of the team, and several other learners including: residents, interns, medical students who are all at various stages of learning. Of course at the center of it all, is the patient and that's you! We want you to step into the role as a patient and offer your perspectives.

We will now introduce a teaching framework on how to have a conversation about this topic with learners, and ask you to think about whether this is feasible to do with patients present as well.

The framework is for all patients with the assumption you have received excellent care from the provider. We want to know your thoughts on the curriculums usage while you are the patient at the bedside. The Physician and Learners would communicate in the form of the 5 Step Teaching Framework.

[Introduce 5-Minute Moment for Racial Justice Teaching Framework]

Facilitator Asks:

- What do you think about this framework and your doctor teaching it to their learners with you in the room?
- What is missing?
- What else would you want doctors-in-training to learn about race and health or caring for patients?

Next, we will introduce a few cases that are written in the structure of this framework and we will ask for your feedback.

Case # 1 Kidney Health and Kidney Disease Case [Post written slides]
A doctor is caring for a 65-year-old Black woman who was hospitalized for heart failure. She also has diabetes, high blood pressure, and long-term kidney disease. After several days of treatment, she is ready to go home.

The resident says to the doctor, “I thought about referring her to a kidney specialist in case she needs dialysis later, but based on her *race-corrected eGFR* (a test that measures kidney health), her numbers don’t look too bad. I think we can wait.”

Question:
How might the use of race in this test be affecting the medical decision-making in her care? Dr. Kidney, the attending physician, steps in and remembers what she learned from the *5-Minute Moment Curriculum.* She takes a moment to include both the patient and her trainees in a short but clear explanation of how racial bias might be affecting this diagnosis.

See what she shares with them next.

Current Standard:
Right now, kidney function test results are often *adjusted for race* in Black patients, but not for people of other races. This means that Black patients’ kidney numbers are shown as about 21% higher than those of non-Black patients.

Background:
About 20 years ago, researchers found it harder to estimate kidney function in Black patients. To fix this, they added a “race correction” to the test formula. They thought this difference was because Black people had more muscle mass than white people—but no study actually measured muscle mass to prove this.

Resulting Difference:
Because the test adds 21%, Black patients often appear to have healthier kidneys than they really do. This overestimate can delay care—Black patients are three times more likely to develop kidney failure and, on average, wait 77 days longer for a kidney transplant compared to white patients.

Steps Toward Equity:
Many hospitals and labs have stopped using the race correction factor when calculating kidney function. Instead, they now use other blood tests that give a more accurate picture of kidney health. These tests help doctors safely dose medications and make the right treatment decisions—without relying on "race norming."

CAB Questions:

- What are your initial thoughts and impressions if you were to hear this information from your doctor?
- What could your doctor say or do to invite your story and contribution into the teaching moment?

Case 2: Diagnosing Skin Findings in Patients with Darker Skin
Facilitator: Next, we will watch a video clip of an educator teaching the learner about health disparities in diagnosing skin conditions across different skin types. Video demonstration: 5mmracialjustice.stanford.edu Video: Diagnosing Skin Findings in Patients with Darker Skin.

- What are your initial thoughts and impressions if you were to hear this information from your doctor?

- What could your doctor say or do to invite your story and contribution into the teaching moment?

- Is there anything else you would want the doctor to bring up during the teaching moment?

- If the doctor were to talk about this topic in front of you with learners, do you feel it would take the attention away from you or your medical care?

Other questions to prompt CAB members: (if time permits)
- What do you think about the framework?

- What is missing from the framework?

- What else would you want doctors-in-training to learn about race and health or caring for patients?

- Now imagine that you are the patient and the Attending Doctor begins to share information to his learners about how race could be related to your health. How would this make you feel?

- Would you be comfortable with the doctor sharing this information while you are present?
